# Soil Carbon Dynamics Reshaped by Ancient Carbon Quantification

**DOI:** 10.1111/gcb.70482

**Published:** 2025-09-02

**Authors:** Yoann Copard, Christine Hatté, Lauric Cécillon, Yannick Colin, Pierre Barré, Claire Chenu, Sophie Cornu

**Affiliations:** ^1^ Univ. Rouen Normandie, Université Caen Normandie, CNRS, Normandie Univ. Rouen France; ^2^ Silesian University of Technology, Institute of Physics Gliwice Poland; ^3^ Laboratoire Des Sciences du Climat et de L'environnement, UMR 8212 CEA CNRS UVSQ, Université Paris‐Saclay Gif‐sur‐Yvette France; ^4^ Univ. Rouen Normandie, INRAE, Normandie Univ. Rouen France; ^5^ Laboratoire de Géologie, Ecole Normale supérieure, CNRS, PSL Univ. Paris France; ^6^ Université Paris‐Saclay, INRAE, AgroParisTech, UMR EcoSys Palaiseau France; ^7^ Aix Marseille Univ., CNRS, IRD, INRAE, Coll France, CEREGE Aix en Provence France

**Keywords:** carbon turnover time, meta‐analysis, parent material, radiocarbon, radiocarbon‐free organic carbon, soil organic carbon

## Abstract

Soil is a major terrestrial carbon reservoir, and enhancing its carbon stock is a central strategy to mitigate climate change. Earth system models project a net soil carbon sink by 2100, the magnitude of which is still under debate, differing significantly between approaches. Radiocarbon‐based studies often suggest a limited soil carbon accumulation capacity, but these estimates are biased by the presence of ancient, radiocarbon‐free, organic carbon (aOC). This carbon no longer contributes to soil carbon dynamics and increases the average ^14^C age of soil carbon because it is radiocarbon‐depleted. This known radiocarbon caveat can be overcome with a better understanding of the aOC (ancient radiocarbon‐free OC) distribution in the world's soils. Here we apply a mixing linear equation to 313 soils worldwide from radiocarbon databases to estimate the aOC contained in soils. The aOC contained in soils has different origins, from rock‐derived to old biospheric C strongly associated with mineral particles during pedogenesis. Our findings show a mean aOC content of 2.4 mg/g ±3.2 SD with an aOC contribution up to 11% of the soil organic carbon in topsoils (0–30 cm depth), reaching 25% in subsoils (30–100 cm depth) and more than half in deep soil (> 100 cm depth). We demonstrate that the aOC content is particularly high in Andosols and Cryosols. We subtracted the aOC contributions to calculate a global mean corrected age of non‐aOC carbon to 1 m depth of 290 years, contrasting sharply with previously reported values of 3100 to 4830 years. This corrected estimate aligns more closely with independent isotopic proxies (^13^C and ^36^Cl) of soil carbon dynamics. These results also reconcile empirical data with the parameterization of Earth system models.

## Graphical abstract

The knowledge of soil organic carbon (SOC) time turnover, in order to estimate the amount of additional carbon that can be stored in soils, is a key point to elaborate a strategy to mitigate climate change. Many studies dealing with this issue use radiocarbon measurements but do not consider the occurrence of ancient organic carbon depleted in radiocarbon, which can increase the mean SOC age. Based on 313 worldwide soil profiles, this work proposes an assessment of the contribution of this ancient carbon and rejuvenates the mean age of SOC, with a possible impact on the potential carbon storage in soils.

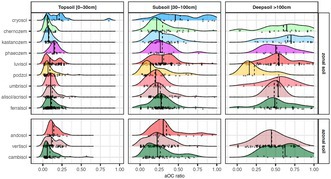

## 1 Introduction

Since soils are the largest terrestrial carbon reservoir (Friedlingstein et al. 2023), positive or negative changes in soil organic carbon (SOC) stocks have an impact on atmospheric CO_2_ concentration over short time steps. It is hardly surprising, therefore, that sustainable policies, such as the 4 per mil initiative launched during COP 21, target their efforts on increasing the carbon sequestration capacity of this key reservoir. However, several grey areas persist in our understanding of SOC dynamics and, as a result, the evolution of SOC stocks remains a major source of uncertainty in climate projections over the 21st century. In particular, mean residence times and mean ages of SOC pools in topsoils but above all in deepsoil layers are still actively discussed (Balesdent et al. 2018; Carvalhais et al. 2014; Grapeloup et al. 2023). It is of primary importance to better constrain the residence time of SOC pools in order to quantify the influence of soils on climate regulation since long‐term storage carbon—reflected in its age—is what defines true sequestration, as opposed to short‐term accumulation that may quickly return to the atmosphere.

Some studies revisited the SOC mean age from 0 to 100 cm depth using Δ^14^C values from globally distributed soil profiles (Shi et al. 2020; He et al. 2016). They conclude that the soil carbon age is far older, by a factor of 6, than predicted by Earth System Models (ESMs) resulting in a slower turnover carbon pool (Lawrence et al. 2019; Zhu et al. 2019) than classically proposed by ESMs. Integrating this radiocarbon‐based constraint in ESMs would imply a severe decline by 40% ± 27% of the potential sequestration of carbon in soils for the 21st century (He et al. 2016). Conversely, a meta‐analysis of soil stable carbon isotope signatures (Balesdent et al. 2018) and a new approach based on ^36^Cl to assess soil carbon dynamics (Grapeloup et al. 2023) proposed an SOC age close to those simulated by ESMs. Additionally, using a global radiocarbon database (ISRaD), a recent study (Grant et al. 2023) alerted the scientific community to the need to consider the impact of ancient radiocarbon‐free organic carbon (aOC) in soils, assumed to be rock‐derived, that increases the apparent SOC residence time. The study showed that since this aOC fraction is radiocarbon‐free, it artificially increases the SOC residence time in soils. In subsoils, Grant et al. (2023) used a mixing model and calculated a correct ^14^C age of SOC by considering two mass fractions of rock‐derived aOC (0.10 or 0.38). The consequence is a rejuvenation of the apparent SOC ^14^C age, initially 3970 ^14^C years (Shi et al. 2020) to a new ^14^C age involved in carbon dynamics of 3100 and 150 ^14^C years depending on the aOC mass fraction considered. However, (i) soils can develop in surficial deposits and recent sediments containing organic carbon of various origins and (ii) aOC is not only rock‐derived but may also originate from long pedogenesis as Ferralsols develop on a timeframe longer than 60 kyr (i.e., radiocarbon‐free).

**FIGURE 1:**
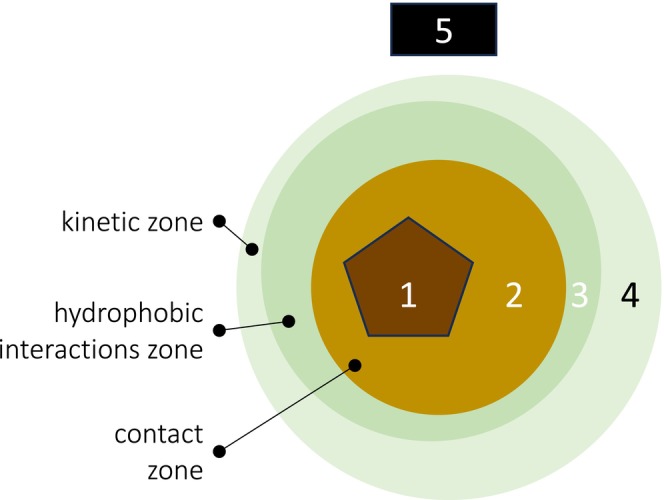
Distribution of aOC originating from both the parent material and pedogenesis. 1: Mineral particles (clays or carbonates, hydrous Fe/Al oxides) zone. This zone is assumed to contain aOC inherited from all sedimentary rocks, possibly low‐grade metamorphic rocks, and sedimentary/surficial deposits containing sedimentary rock fragments (e.g., alluvium, colluvium, glacial deposits). This aOC fraction is called rock‐derived aOC and has a F^14^C = 0. Zones 2 to 4 are based on the model of Kleber et al. (2007) for SOC originating from pedogenesis. 2: Contact zone containing aged organic molecules strongly linked to mineral particles. Since the SOC contained in this zone is of biospheric origin and strongly linked to mineral particles, we consider it as SOC with F^14^C > 0 but close to 0 like the SOC from loess. 3–4: Hydrophobic and kinetic zones containing organic molecules that are fully involved in soil carbon dynamics. We consider that the SOC, younger than that located in Zone 2, in these zones has F^14^C > 0 and that the more peripheral the molecules are, the younger they are. 5: Particulate organic matter showing various F^14^C from modern (leaves, organisms with F^14^C ≃ 1.00) to poorly reactive organic particles such as charcoals that can be very aged and could also be associated to other organic molecules such as mineral particles.

Considering strictly rock‐derived aOC, numerous case studies have clearly reported that aOC contributes up to 50% of the SOC (Petsch et al. 2001; Copard et al. 2006; Kalks et al. 2021; Reichenbach et al. 2021; Grant et al. 2023). At a global scale, 43 Tg y^−1^ of aOC resulting from the weathering of sedimentary rocks could feed the deep soil (Copard et al. 2007). This rock‐derived aOC, constituting the kerogen, is chemically (ligand exchange and adsorption) or physically (entrapment of organic molecules) linked to mineral particles such as clays, carbonates, hydrous Fe/Al oxides, and even geological charred particles (Figure 1, Zone 1).

During pedogenesis, a fraction of SOC is strongly involved in organo‐mineral interactions. This close interaction of SOC with mineral surfaces has been theorized by Kleber et al. (2007) and purposely redrawn in Figure 1. In the case of long‐term pedogenesis, part of this organic carbon can correspond to organic molecules of non‐rock‐derived (but biospheric) origin, of low energy and/or not easily accessible to microorganisms. These organic molecules possibly contain SOC that is highly depleted in ^14^C (i.e., with a fraction of modern F^14^C close to 0, Figure 1, Zone 2). The rest of the SOC consists of more peripheral molecules (Figure 1, Zone 3 and 4), derived from the biosphere and showing more or less rapid turnover with higher F^14^C as one approaches the periphery.

**FIGURE 2:**
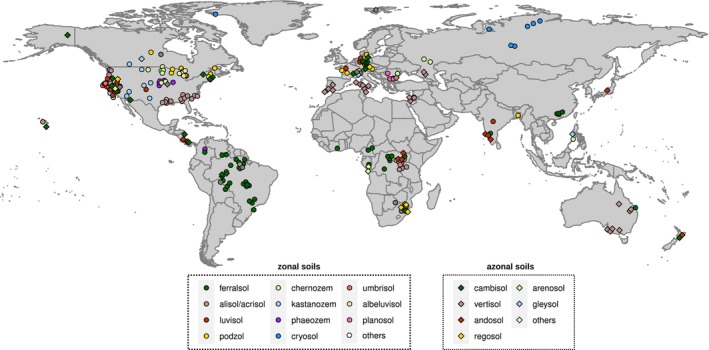
Soil profiles contained in the radiocarbon ISRaD datasets (Lawrence et al. 2020) completed by extra data used in meta‐analysis by Hatté (2022) (hereafter named “LSCE database”): Location and soil classes according to the WRB classification of soil groups.

Using a method previously developed by Galy et al. (2008), also applied by Grant et al. (2023) to soils, we calculated the aOC contents of 2204 soil layers from 313 worldwide‐distributed soil profiles (Figure 2) from the radiocarbon ISRaD datasets (Lawrence et al. 2020) completed by additional abiotic information used in a meta‐analysis (Mathieu et al. 2015; Hatté 2022; hereafter the “LSCE database”). Thanks to this unprecedented dataset, we explored the respective impact of soil and parent material on the aOC mass concentration and on the contribution of aOC in soils. Lastly, we demonstrate how accounting for the amount of aOC changes the average age value of the remaining SOC.

## 2 Materials and Methods

### 2.1 Data Source and Processing

We analysed bulk radiocarbon data from worldwide soil profiles compiled in two databases: the first is the international soil radiocarbon database (ISRaD database; ISRaD_extra data product version v2.0.1. downloaded on 5 April 2022, Lawrence et al. 2020) and the second is the French “Laboratoire des Sciences du Climat et de l'Environnement” (LSCE) database (Hatté 2022). With the exception of 63 profiles, the profiles belonging to the LSCE database are found in the ISRaD one. However, the LSCE database contains more information on abiotic variables (field observations of soil groups, climate variables derived from publications) and an estimate of SOC content originating from loess formation. For both databases, we extracted all the mineral soil layers (defined as soil layers with a measured SOC mass concentration below 120 mg/g of dry weight) with measured fraction modern (the deviation from the ^14^C/^12^C ratio of the Oxalic Acid I SRM 4990B standard; F^14^C). Soil profiles with at least 4 layers distributed over a depth of at least 50 cm were selected. This resulted in 359 soil profiles (ISRaD: 296, LSCE: 63). For each soil layer, we normalized the bulk F^14^C measurement to a sampling year in 2000 (Shi et al. 2020). To do so, we used a time‐dependent, one‐pool steady‐state model of soil carbon dynamics (Gaudinski et al. 2000; Equation 1) fed with a compilation of published time series of atmospheric F^14^C_atm_ measurements for the Northern Hemisphere, the Tropics and the Southern Hemisphere (Hua et al. 2013; Graven et al. 2017; Reimer et al. 2020).

(1) $$F^{14}C_{\mathrm{SOC}}\;\left(t\right)=\sum_{j}^{j-t}\left[F^{14}C_{\mathrm{atm}}.k+F^{14}C_{\mathrm{SOC}\left(j-1\right)}.\left(1-k-\lambda \right)\right]$$

where F^14^C_atm_ and F^14^C_SOC_ are the radiocarbon activities of the atmosphere and of SOC, respectively, reported as fractions of modern, *k* is the decomposition rate of SOC in year^−1^, *λ* is the radioactive decay constant for radiocarbon (ln(2)/5730), *t* is the time (year) for which calculation is being performed (sampling year), and *j* is the time difference between a year (up to 53 kyr) and the sampling year.

We ran this simple model of the soil carbon cycle from −53 kyr to the present at annual time steps, and for SOC turnover times (1/*k*) ranging from 1 year to 50 kyr. Our simulations assumed an equilibrium for SOC stock and constant soil carbon inputs whose radiocarbon signature followed that of the atmosphere. For each sampling year of soil samples, the simulations yielded the relationship between the radiocarbon signature (F^14^C_SOC_) and the turnover time (equivalent to mean age for a one‐pool model) of SOC. We used this set of relationships to infer the turnover time of SOC in each soil layer based on its measured F^14^C_SOC_ value. Then, each bulk radiocarbon signature of a soil layer sampled in year Y (F^14^C_SOC(*Y*)_) was normalized to the year 2000 by selecting the F^14^C_SOC(2000)_ value that corresponded to its inferred turnover time of SOC (Shi et al. 2020). Bulk F^14^C_SOC_(*t*) values equal to or lower than 0.16 (corresponding to an inferred turnover time of SOC higher than 48 kyr, regardless of the sampling year and the geographical position of the soil profile) were not normalized to year 2000.

### 2.2 Quantification of the Ancient Radiocarbon‐Free Carbon Fraction in Soils

#### 2.2.1 Determining aOC by the Linear Model

The radiocarbon activity of each soil layer can be expressed by a binary mixing equation (Galy et al. 2008; Equation 2).

(2) $$F^{14}C_{\mathrm{SOC}}=\left(\frac{\mathrm{aOC}}{\mathrm{SOC}}\right).F^{14}C_{\mathrm{aOC}}+\left(\frac{{\mathrm{SOC}}_{<60\mathrm{kyr}}}{\mathrm{SOC}}\right).F^{14}C_{\mathrm{SOC}<60\mathrm{kyr}}$$

where SOC is expressed as the total mass concentration (expressed in mg/g) of a given layer, F^14^C_SOC_ is the radiocarbon activity of SOC normalized to 2000, aOC is the mass concentration of ancient soil organic carbon (expressed in mg/g), SOC_<60kyr_ is the mass concentration of SOC younger than 60 kyr (expressed in mg/g), F^14^C_aOC_ is the radiocarbon activity of aOC equal to zero (Zone 1, Figure 1). F^14^C_SOC<60kyr_ is the radiocarbon activity of SOC younger than 60 kyr reported as a fraction of modern and belonging to Zone 3 and 4 (see details in Figure 1).

Equation (2) can be rearranged and simplified by: (i) injecting F^14^C_aOC_ = 0, (ii) multiplying the terms by SOC (Equation 3), (iii) inserting SOC_<60kyr_ = SOC—aOC in Equation (3) (Equation 4), and (iv) replacing the variable from Equation (4) as follows: *Y* = SOC, F^14^C_SOC_ and *X* = SOC (Equation 5, Galy et al. 2008).

(3) $$\mathrm{SOC}.F^{14}C_{\mathrm{SOC}}={\mathrm{SOC}}_{<60\mathrm{kys}}.F^{14}C_{\mathrm{SOC}<60\mathrm{kyr}}$$

(4) $$\mathrm{SOC}.F^{14}C_{\mathrm{SOC}}=F^{14}C_{\mathrm{SOC}<60\mathrm{kyr}}.\left(\mathrm{SOC}-\mathrm{aOC}\right)$$

(5) $$Y=F^{14}C_{\mathrm{SOC}<60\mathrm{kyr}}.\left(X-\mathrm{aOC}\right)$$

We used this linear model to estimate the mass concentration of the aOC carbon component (expressed in mg/g).

For each soil layer, the aOC proportion was then calculated as described in Equation (6).

(6) $$\mathrm{aOC}\;{\text{ratio}}_{i,j}=\frac{{\mathrm{aOC}}_{i}}{{\mathrm{SOC}}_{i,j}}$$

where aOC ratio is the aOC proportion in the soil layer *j* of the soil profile *i* (unitless), aOC_i_ is the mass concentration of ancient SOC in the soil profile *i* (expressed in mg/g), and SOC_*i,j*_ is the total SOC mass concentration in the soil layer *j* of the soil profile *i* (expressed in mg/g).

#### 2.2.2 The Particular Case of Loess

Soils developed in surficial sedimentary formations—such as loess, alluvial deposits, or colluvium—differ fundamentally from those formed directly on hard bedrock. These surficial formations have undergone various phases of erosion, transport, and deposition, and thus incorporate carbon from multiple sources. Among them, loesses represent a particular case, not only because their genesis is unique, but because they are one of the few superficial deposits for which independent data exist to characterize and isolate intermediate‐age carbon contributions.

In the case of loess‐derived soils, three distinct carbon sources can be identified: (1) a petrogenic pool (aOC, Zone 1 in Figure 1) inherited from the mineral dust prior to loess deposition, (2) a glacial‐age biospheric pool with low F^14^C values depending on the age of the loess (typically from 25 to 15 kyr, Zone 2 in Figure 1) associated with the vegetation that stabilized the dust and facilitated the accumulation of typical loess deposits, and (3) a biologically active soil carbon pool, reflecting ongoing biological inputs and pedogenic processes, characterized by higher F^14^C values (Zone 3 and 4 in Figure 1). Thanks to existing paleoclimatic studies on nearby loess sequences (e.g., Antoine et al. 1999; Crouvi 2024), it was possible to estimate the radiocarbon signature and organic carbon mass concentration of the glacial biospheric pool. This allowed us to subtract its contribution from the SOC signal, isolating only the aOC and active contributors—an approach not feasible for most other surficial sediments due to the lack of comparable reference data. Then, these last two sources with their respective values follow the series of equations (Section 2.2.1).

#### 2.2.3 Hypothesis, Limit of Quantification and Accuracy of the Model

**FIGURE 3:**
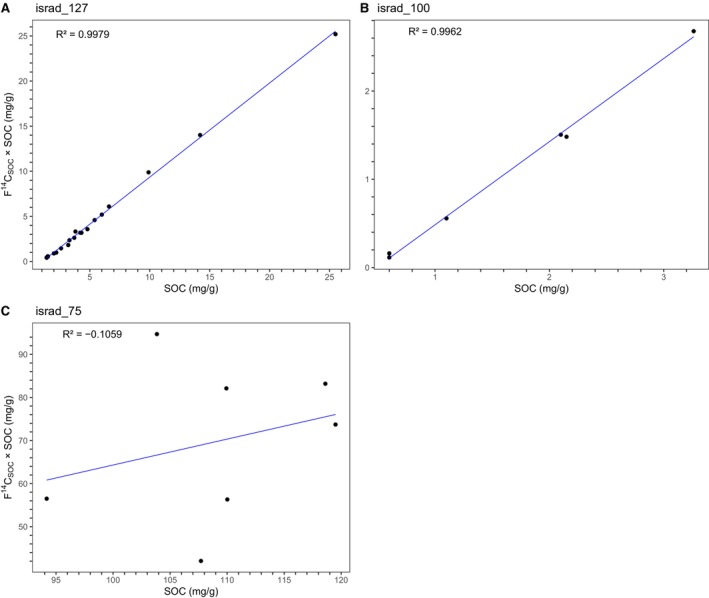
Examples of linear modeling derived from Galy et al. (2008) of soil profiles from the ISRaD database showing various *R* ^2^. (A) Krull and Skjemstad (2003) (CHI profile, 30 layers), (B) Horwarth (2007) (6 layers), (C) Giardina et al. (2014) (6 layers). With coefficient correlations over 0.99, profiles A and B meet our conditions while profile C is excluded from our analyses.

If we consider a constant concentration of aOC for a given soil profile, this concentration corresponds to the value of *X* for *Y* = 0 (Equation 5). This hypothesis is reasonable for soil profiles developed in a vertically homogeneous parent material. The aOC fraction can be considered as equivalent to the IOM pool defined in SOC dynamic models as RothC (Jenkinson and Coleman 2008). These authors showed that it varied only slightly with depth, and it is therefore classically considered as constant with depth in the literature (e.g., Jagercikova et al. 2017). Poor linear relations could be due to pedogenesis developed in various parent materials or a polyphase pedogenesis leading to aOC able to be processed (Figure 3, Figure S1). Lastly, for seven profiles (out of 313), negative values of aOC were obtained. Negative aOC values have no physical meaning and were thus set at zero.

Since aOC values considered as null can result in negative values by the model, we considered the median value of these negative aOC estimates as the limit of quantification of the model. This value was set at −0.11 mg/g. To estimate the standard deviation (SD) on the content of ancient SOC in a given soil profile, we injected the standard deviations provided by the regression analysis of the intercept, − F^14^C_SOC<60kyr_. aOC, and of the slope, F^14^C_SOC<60kyr_, in Equation (5). For each soil profile, the coefficient of determination (*R* ^2^) of the simple linear regression model was used as a quality criterion for its aOC content. Soil profiles with models showing *R* ^2^ > 0.9 and an aOC ratio < 1 (see below) were selected for further analysis (Figure 3, see also examples in Figure S1).

As a result, 313 soil profiles (261 ISRaD and 52 LSCE) containing 2204 soil layers were retained in this study, among which 253 were classified according to the WRB soil classification.

### 2.3 Calculation of the Mean Age of Soil Organic Carbon

Radiocarbon‐based studies of SOC dynamics usually calculate the mean age of total SOC that is present in a soil sample or a soil fraction as follows (Equation 7, Torn et al. 2009).

(7) $${\mathrm{Age}}_{\mathrm{SOC}}=-\left(\frac{5\;730}{\ln\;\left(2\right)}\right)\times \ln\left(F^{14}C_{\mathrm{SOC}}\right)$$

where Age_SOC_ is the mean age of total SOC (expressed in years), 5730/ln(2) is the mean life of radiocarbon, and F^14^C_SOC_ is the radiocarbon activity of the soil layer reported as a fraction of modern (normalized to the year 2000 when raw values were higher than 0.16, see above).

We used Equation (8) to calculate the radiocarbon activity of the recent component of SOC by considering the dilution by aOC.

(8) $$F^{14}C_{\mathrm{SOC}<60\mathrm{kyr}}=\frac{F^{14}C_{\mathrm{SOC}}}{\left(1-{\mathrm{aOC}}_{\text{ratio}}\right)}$$

where F^14^C_SOC<60kyr_ is the radiocarbon activity of the component of SOC younger than 60 kyr reported as a fraction of modern, F^14^C_SOC_ is the radiocarbon activity of the total SOC reported as a fraction of modern, and aOC_ratio_ is the proportion of aOC with a F^14^C_aOC_ = 0.00.

We then combined Equations (7) and (8) to calculate the mean age of the component of SOC younger than 60 kyr in the soil layers (Equation 9).

(9) $${\mathrm{Age}}_{\mathrm{SOC}<60\mathrm{kyr}}=-\left(\frac{5\;730}{\ln\;\left(2\right)}\right)\times \ln\left(F^{14}C_{\mathrm{SOC}<60\mathrm{kyr}}\right)$$

where Age_SOC<60kyr_ is the mean age of the component of SOC younger than 60 kyr (expressed in ^14^C year).

## 3 Results

### 3.1 Ancient Radiocarbon‐Free Organic Carbon Is Widespread in Soils

**FIGURE 4:**
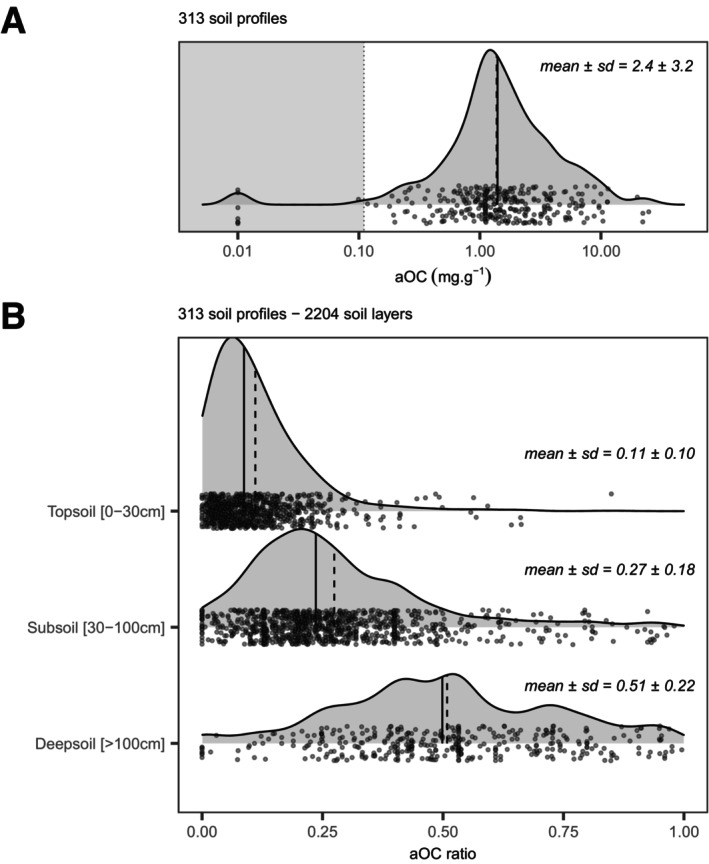
Densiplots of (A) Global mass concentration of aOC (mg/g) in soils from the radiocarbon ISRaD and LSCE datasets; and (B) AOC ratio (aOC/SOC) in top (0–30 cm), sub (30–100 cm) and deep soils (> 100 cm). Note the logarithmic scale for the *x*‐axis in A. The vertical dashed and plain lines stand for the average and median values. The gray area (A) corresponds to the limit of quantification (0.11 mg/g).

The 313 worldwide‐distributed soil profiles (Figure 2) exhibit a mean aOC mass concentration of 2.4 ± 3.2 mg/g (Figure 4A). The aOC proportion (aOC ratio) increases from low values in topsoils (0–30 cm depth, 0.11 ± 0.10) to significantly higher values in subsoils (30–100 cm depth, 0.27 ± 0.18) and deepsoils (> 100 cm depth, 0.51 ± 0.22) for all the soil profiles (Figure 4B). This expected trend is due to the input of OC from plants via above‐ and below‐ground litter, which decreases with soil depth. At the global scale, with respect to the estimate of SOC stocks (Batjes 1996; Jobbagy and Jackson 2000), topsoils hold 704 Gt of OC, of which 77 Gt could be aOC; in subsoils, this global OC stock reaches 801 Gt, of which 216 Gt may be aOC, while deeper (between 1 and 2 m depth), the OC stock is close to 910 Gt, of which 464 Gt is aOC.

As mentioned in the introduction, aOC can originate from the parent material or from pedogenesis. To explore this, we analyzed all aOC data according to soil groups and parent material types for groups or types with more than 4 profiles. Twelve soil groups (Cryosols, Chernozems, Kastanozems, Phaeozems, Luvisols, Podzols, Umbrisols, Alisols/Acrisols, Ferralsols, Andosols, Vertisols, and Cambisols) corresponding to a surface of almost 52% (66.65 Mkm^2^) of the global land surface without snow cover (www.isqper‐is.eu, www.isric.org, Table S4) and 7 types of parent material (surficial deposits, sedimentary deposits, sedimentary rocks, loess, volcanic rocks, plutonic rocks, and metamorphic rocks) were thus considered (Table S1).

**FIGURE 5:**
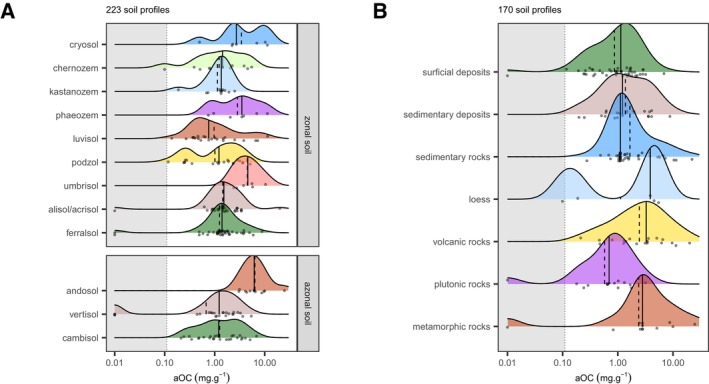
aOC mass concentration (in mg/g) for: (A) the different soil groups of the WRB classification encountered in the radiocarbon ISRaD and LSCE datasets. The soil classes were separated into zonal soils, whose development is mainly due to climate, and azonal soils, whose development is mainly due to other soil‐forming factors (Jenny 1941) such as nature of the parent material (Vertisols, Andosols) or time (Cambisols); and (B) the main parent material types excluding Cryosols and Ferralsols. Note the logarithmic scale for the *x*‐axis. The vertical dashed and plain lines stand for the average and median values. The gray zone corresponds to aOC values lower than the limit of quantification.

**FIGURE 6:**
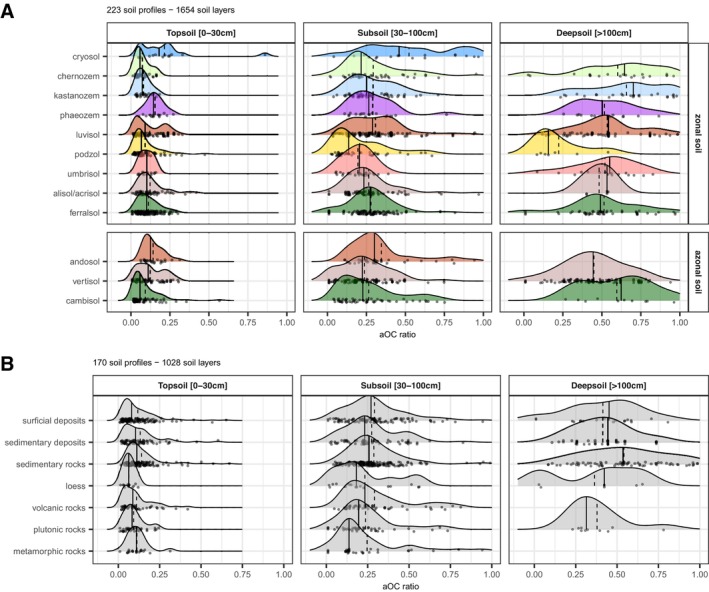
The contribution of aOC in top, sub, and deep soils: (A) of WRB soil groups and (B) of selected parent materials. The soil groups were separated into zonal soils, whose development is mainly due to climate, and azonal soils, whose development is mainly due to other soil‐forming factors (Jenny 1941) such as the nature of the parent materials (Vertisols, Andosols) or time (Cambisols).

Ferralsols, Acrisols/Alisols, Umbrisols, Andosols, and Kasternozems have a normal aOC distribution. A mass concentration of 7.5 ± 5.7 mg/g is estimated for Andosols (*n* = 13) higher than that recorded in Umbrisols, which in turn exhibit an aOC content higher than that of Ferralsols, Acrisols/Alisols, and Kasternozems (with 1.3 < mean aOC < 2.3 mg/g, Table 1). In contrast, Cambisols, Chernozems, and Phaeozem present a very large aOC concentration distribution (Figure 5A). For Cambisols, which are poorly developed (young) soils, this large aOC concentration distribution may be due to the large range of parent materials in which they developed. Lastly, Cryosols, Luvisols, Podzols, Vertisols, and Kastanozems exhibit a multimodal aOC concentration distribution with a high aOC concentration mode for Cryosols, Luvisols, and Kastanozems, and a low aOC concentration mode, and a low (in some cases zero) aOC mass concentration mode for Luvisols, Podzols, and Vertisols, respectively (Figure 5A). Interestingly, Cryosols have the highest ratio for both topsoil and subsoil, while Andosols are only richer for the topsoil and exhibit an opposite trend compared to other soil groups (Figure 6A).

**TABLE 1:** aOC concentration reported in mg/g for each soil group according to the WRB classification and associated to the standard deviation within the same soil type.

WRB classification	Profiles number	[aOC] (mg/g)
Azonal soils		
Andosols	13	7.5 ± 5.7
Arenosols	3	0.61 ± 0.03
Cambisols	26	1.9 ± 1.7
Cambisols/Regosols	1	1.7 ± NA
Fluvisols	1	1.0 ± NA
Gleysols	3	2.6 ± 2.3
Regosols	4	0.9 ± 0.6
Stagnosols	2	1.6 ± 0.6
Vertisols	25	1.5 ± 1.3
Zonal soil		
Albeluvisols	4	3.9 ± 3.5
Alisols/Acrisols	30	2.3 ± 3.5
Calcisols	1	0.6 ± NA
Chernozems	12	2.0 ± 1.8
Chernozems/Kastanozems/Phaeozems	2	1.6 ± 1.2
Cryosols	7	5.2 ± 4.3
Ferralsols	41	1.9 ± 1.7
Histosols	2	11.2 ± 15.5
Kastanozems	12	1.3 ± 0.6
Luvisols	23	1.9 ± 2.8
Nitisols	2	1.0 ± 0.4
Phaeozems	7	4.2 ± 3.8
Planosols	3	2.0 ± 0.4
Plinthosols	1	0.9 ± NA
Podzols	21	1.6 ± 1.4
Solonetz	1	0.17 ± NA
Umbrisols	6	5.1 ± 2.9

*Note:* The soil groups were separated into zonal soils, whose development is mainly due to climate, and azonal soils, whose development is mainly due to other soil‐forming factors (Jenny 1941) such as nature of the parent material (Vertisols, Andosols) or time (Cambisols). The number of profiles for each soil group is also reported.

The mean aOC concentrations also vary as a function of the parent materials (Figure 5B, Table 2). However, many studies do not mention or remain evasive in their description of the nature of the material in which the studied soils are developed. As a result, in one‐third of cases it was not possible to determine the nature of the parent material, while for the two remaining thirds we were only able to classify the parent material into very broad categories (list above, Table S1). Due to the use of these very large categories of parent material, the aOC concentration range encountered in the different parent material categories is very large. Nevertheless, soils developed in volcanic and metamorphic rocks tend to present higher aOC concentrations.

**TABLE 2:** aOC concentration reported in mg/g for each major group of parent material.

Parent materials	Profiles number	[aOC] (mg/g)
Loess	5	2.8 ± 2.5
Metamorphic rocks	12	5.5 ± 6.8
Plutonic rocks	15	0.9 ± 0.8
Sedimentary deposits	21	2.1 ± 2.0
Sedimentary rocks	50	2.6 ± 3.7
Surficial deposits	47	1.3 ± 1.2
Volcanic rocks	20	4.3 ± 4.8

*Note:* These parent materials, checked for each soil profile, comprised 19 categories (Table S1) and were grouped for simplification into 7 major groups. Surficial deposits encompass different environments from glacial to riverine. Sedimentary deposits consist of recent sediments mainly stored in lacustrine and riverine environments.

### 3.2 Change in the Mean Age of SOC Younger Than 60 kyr

Based on the estimated aOC ratio, we recalculated the age of the remaining SOC_<60kyr_ holding ^14^C. The mean ages obtained were 140 ± 570, 420 ± 1230, and 800 ± 2140 years for topsoil, subsoil, and deepsoil respectively (Table 3). These ages are far younger than those calculated by Shi et al. (2020) on the same ISRaD database by a steady‐state one‐pool model with ages of 1390 ± 310 and 8280 ± 2820 years for topsoil and subsoil respectively. Likewise, with a mean age of 290 ± 990 years, our finding for the first 1 m depth is far younger than the reassessment proposed by He et al. (2016) using ^14^C data (3100 ± 1800 years) or by Shi et al. (2020) (4830 ± 1730 years), but lines up with the ESMs (He et al. 2016; 430 ± 50 years).

**TABLE 3:** ^14^C age of SOC without (Age_SOC_) and with (Age_SOC<60kyr_) the contribution of aOC for all the soil profiles according to top, sub, and deep soils of the ISRaD and LSCE databases.

Soil depth	aOC ratio	Age_SOC_ (year)	Age_SOC<60kyr_ (year)
Topsoil (0–30 cm)	0.11 ± 0.10	450 ± 960	14 ± 570
Subsoil (30–100 cm)	0.27 ± 0.18	2340 ± 2970	420 ± 1230
Deepsoil (> 100 cm)	0.51 ± 0.22	5300 ± 3450	800 ± 2140

*Note:* aOC ratio is given for information.

## 4 Discussion

### 4.1 Origin of the Ancient Organic Carbon Distribution in Soils

#### 4.1.1 Long Pedogenesis‐Derived aOC in Soils

Cryosols, mainly from Siberia in the dataset, exhibit a large aOC mass concentration mainly due to the inherited OC incorporated in soils over several climatic cycles and stabilized during the Pleistocene (Biller‐Celander et al. 2021; 400 kyr). This long‐term storage of SOC, due to cold climatic conditions, explains why the aOC proportion in these soil profiles is high. Global warming could nevertheless make this dormant reactive aOC available in fluxes between C reservoirs (Windirsch et al. 2020; Strauss et al. 2017).

Ferralsols are also well‐known to experience long pedogenesis (Lepsch and Buol 1974). Nevertheless, they contain an aOC proportion close to the global average of worldwide soils (Figure 6A, Table 1), meaning a slow accumulation of aOC during pedogenesis due probably to the high reactivity of SOC under this warm climate and a low initial aOC proportion in the parent material. Indeed, sedimentary rocks, sedimentary deposits, and plutonic rocks, which contain a low amount of aOC (Figure 5B), were the most frequent parent materials encountered for the Ferralsol profiles analyzed when information was available (two‐thirds of the cases).

For these two soil groups (Cryosols and Ferralsols), aOC can be interpreted as resulting at least partly from a biospheric (pedogenetic) origin and not from a rock‐derived origin, with contrasting aOC concentrations explained by the contrasted climates under which these two soils developed. Since we considered that in Cryosols and Ferralsols, aOC is mainly derived from the pedological history, these soils were excluded from the analysis of the importance of the parent material on the aOC concentration.

#### 4.1.2 Parent Material‐Derived aOC in Soils

Whatever the nature of the parent material of the soil profiles, aOC concentrations and hence proportions are never null or close to the limit of quantification (i.e., 0.11 mg/g; Figures 5B and 6B, Table S2). Many soils develop on surficial and sediment deposits and not on the underlying geological formation reported in global geological maps (Dürr et al. 2005; Hartmann and Moosdorf 2012). For that reason, unlike previous studies (Grant et al. 2023), we did not use this source of information to assess the soil parent material when not reported in the study. Surficial and sediment deposits inherit from a large variety of rocks to which they are directly spatially connected when autochthonous (non‐significant lateral transport) or conversely disconnected when allochthonous (significant lateral transport within the watershed). This could explain the wide distribution of aOC concentrations (Figure 5B; Table 2; surficial: 1.3 ± 1.2 mg/g; sediment: 2.1 ± 2.0 mg/g). Accordingly, these deposits contain aOC inherited from a more or less complex history with various aOC origins: (i) from the rocks from which they derived, especially if these rocks are sedimentary and to a lesser extent metamorphic and (ii) from a previous pedogenesis before partial resetting by sedimentary transport. Therefore, aOC can be located in Zone 1 (if rock‐derived aOC) or in the contact Zone 2 of the adapted Kleber model (Figure 1) in the case of aOC derived from previous pedogenesis. A typical example of this is loess deposits. As their age and initial SOC concentration are available in the literature, a correction can be applied on loess OC. Despite an age ranging from 10 to 20 kyr, our results show that the soil profiles developed on loess also contain a fraction of aOC equal in average to 2.8 ± 2.5 mg/g (Table 2).

Sedimentary rocks cover almost 2/3 of continental surfaces (Dürr et al. 2005). As they have been shown to contain aOC, their weathering contributes rock‐derived aOC to the soils, as observed in many studies (Copard et al. 2007; Petsch 2014 with references therein, Grant et al. 2023; Evans et al. 2025). Our results show that soils developed on sedimentary rocks contain 2.6 ± 2.0 mg/g of aOC (Figure 5B, Table 2). Interestingly, this aOC concentration is close to that of the sedimentary rocks themselves, for instance limestones (2.8 mg/g) or sandstone (2.4 mg/g in Ronov and Yaroshevskiy 1976), and thus probably inherited from the weathering of the parent material (Copard et al. 2007; Evans et al. 2025).

As stated above, plutonic, volcanic, and most metamorphic rocks are well known to be devoid of rock‐derived aOC. Whilst the lowest aOC concentrations were recorded for soils developed on plutonic rocks, they remain surprisingly significant for such rocks (0.9 ± 0.8 mg/g, Table 2). The weathering products of the silicates that compose them may have a binding energy responsive to strong organo‐mineral interactions (Kleber et al. 2007; contact Zone 2). Hence, non‐rock‐derived aOC could be trapped for long periods with a low energy content available for microbial biomass (Lehmann and Kleber 2015). Soils developed on metamorphic rocks had the highest aOC content (5.5 ± 6.8 mg/g, Figure 5B, Table 2), with some soils having no aOC (i.e., under the limit of quantification). Indeed, while metamorphic rocks (e.g., gneiss, micaschists, amphibolite) are devoid of rock‐derived aOC, some of them, such as schists or graphitic phyllites, exhibit high OC contents (possibly exceeding 50 mg/g, Thapa et al. 2023), explaining the occurrence of such high aOC concentrations in soils. However, more information on the soil parent materials is needed to confirm this hypothesis.

#### 4.1.3 Lithospheric/Mantellic aOC Origin in Soils (Andosols)

Soils developed on volcanic rocks exhibit aOC mass concentrations that are extremely variable and, on average, surprisingly high for soil developed on rocks without aOC (4.3 ± 4.8 mg/g, Figure 5B, Table 2), leading to a significant contribution of aOC in the topsoil and subsoil (Figure 6B, Table S3). Among these soils, Andosols are by far richer in aOC (7.5 ± 5.7 mg/g, Figure 5A, Table 1), with a significant aOC contribution (Figure 6A) in both the topsoils (0.13) and subsoils (0.34). Since there is no rock‐derived aOC in these volcanic rocks, two hypotheses can be proposed. The first corresponds to the case of plutonic and metamorphic rocks for which the aOC is inherited from a long pedogenesis with strong organo‐mineral interactions classically observed in volcanic soils (Grant et al. 2022), notably in Andosols (e.g., de Junet et al. 2013). The second hypothesis, nonexclusive of the former, is that locally, these soils developed near volcanoes degassing radiocarbon‐free mantellic/lithospheric CO_2_. This radiocarbon‐dead CO_2_ is absorbed by the surrounding vegetation via photosynthesis (Pasquier‐Cardin et al. 1999) and released to the soils by the root exudates and the litter, resulting in recent OC devoid of radiocarbon in dynamic organic molecules (Figure 1, Zone 3 and 4).

As a partial conclusion, while our results point to the importance of soil parent material to explain this aOC contribution to SOC dynamics, parent materials are often poorly described in the literature. In order to reinforce the robustness of our findings from these WRB soil groups covering 52% of the total land surface, future work should focus (i) on the other 14 WRB soil groups not considered in this study, covering almost 1/3 of the land surface (e.g., Arenosols, Calcisols, Gleysols, Regosols, Fluvisols), by densifying the sampling of profiles and by performing radiocarbon measurements, and also (ii) on other soil groups (e.g., Leptosols, Lixisols, Solonchaks, Durisols, Retisols and Gyspisols) for which we have no information in the two databases.

### 4.2 aOC Quantity Strongly Modifies the Mean Age of SOC

The robustness of our age correction and the consideration of aOC in soil profiles is supported by comparing the age proposed for Ferralsols. For Ferralsols, we calculated an age of 25 ± 290 years for the topsoils, 300 ± 910 years for the subsoils, and 330 ± 1080 years in the deeper part of these soils (Table S2). These ages echo those calculated independently with the use of 55 C3‐C4 chronosequences (180 and 440 years, Balesdent et al. 2018) while some mean ages of 390 years (topsoils) and 2970 years (subsoils) were found for tropical forest soils (Shi et al. 2020). Similarly, our correction at least partially captures the contribution of aOC to the Cryosol carbon pool and shows a mean age of the reactive carbon of 240 ± 480 (topsoils), 490 ± 890 (subsoils) and 590 ± NA years (deep soils, Table S2), much younger than already existing ages provided for soil profiles at high latitude for which some extremely old ages were found in subsoils (16,890 and 15,440 years, Shi et al. 2020). Not surprisingly, the recalculated age for Andosol topsoils is also rejuvenated (80 ± 260 years rather than 370 ± 540 years without this correction) as seen for Andosol subsoils (670 ± 1930 years instead of the 3170 ± 4220, Table S2).

With 77 Gt aOC in topsoil (11% of the stock), 216 Gt aOC in subsoil (27% of the stock) and 464 Gt aOC in deepsoil (51% of the stock), the mean SOC age calculated by radiocarbon must be corrected for the significant presence of aOC. Without this correction, this mean age is therefore strongly overestimated.

### 4.3 Foreseeable Considerations and Future Research Directions of aOC in Soils

The aim of this study was to solve the discrepancy existing in the literature between ^14^C derived ages for SOC and isotopic/ESM ages. The hypothesis was that this discrepancy was due to the integration of an ancient OC fraction, inherited from the parent material, and thus not active in the SOC cycle, in the estimate of the SOC age. To do so we used a two‐compartment mixing model. We also showed that for surficial and sediment deposits having multiple origins (e.g., the complexity of the sedimentary cascade in a watershed), a combination of different sources is probable and should be appraised by a more complex model (e.g., three‐compartment for loess). Due to the lack of available data, only loess formations were modeled with such an approach as they have a known age and OC mass concentration. For other surficial/recent sediment deposits, using site‐specific F^14^C values or recognizing that some ancient C may have a small, but measurable, ^14^C could refine estimates. This would require new data for these surficial and sediment deposits. In addition, while SOC dynamic modeling using SOC pools with different mean turnover times is a classical approach in the literature (Roth C and Century), it is now well admitted that the SOC exhibits a continuum of ages. Therefore, the SOC ages provided are only mean ages and should be considered as such.

It should be mentioned that an aOC‐like compartment is considered in SOC dynamic modeling such as in Roth C (Jenkinson and Coleman 2008), in which an Inert Organic Matter (IOM) pool is defined, or in Century (Parton et al. 1987) in which a passive SOC pool, including inert SOC, with a mean residence time of several centuries is used. Our analysis of the aOC shows that this compartment should be allowed to grow over time, especially when long‐term simulations are considered, as the aOC is not only inherited from the parent material but also builds up during a long pedogenesis. Indeed, aOC that is poorly mobilized by microorganisms since it is embedded in molecules with a low energy content (e.g., rock‐derived aOC and inherited aOC‐like from loess), and aOC that is derived from long or previous pedogenesis and that can be mobilized over a long timescale, may have different dynamics (from totally inert to a slow turnover). This aOC quantification and contribution will provide a more realistic view of how the SOC dynamics need to be considered in ESMs and will reinforce the SOC turnover proposed in such global models (e.g., Todd‐Brown et al. 2014; Luo et al. 2016).

While aOC is more recalcitrant in soils than the downward input of biospheric SOC, the microbial decomposition of part of this aOC cannot be excluded: this decomposition is a function of climate and land use (Evans et al. 2025) especially in harsh climatic conditions (Petsch 2014 and references therein) or in the event of a rapid exhumation of rocks (Hemingway et al. 2018). As an example for tropical soils, a study clearly demonstrated that although half of the SOC was composed of aOC in the subsoil, this aOC can nevertheless be significantly reactive (Reichenbach et al. 2021). Ultimately, this aOC can join a faster cycling soil C pool by entering the soil food chain (e.g., Petsch et al. 2001).

To explain this aOC contribution and dynamics in soils, future work should consider the whole geological history imprints of soil parent material, which could be coupled to other external parameters (e.g., temperature, humidity, and land‐use history may enhance the weathering rates of aOC) and the reactivity of aOC in soils, integrating the different aOC origins (parent material including rocks and surficial/sediment deposits, soil experiencing long pedogenesis), to draw a complete picture of the global C cycle involving the exogenous aOC cycle (Blattmann 2022; Evans et al. 2025). For soils, this will be a crucial challenge as this C reservoir could contribute to mitigating the rise of atmospheric C.

## Author Contributions

**Yoann Copard:** conceptualization, formal analysis, methodology, supervision, writing – original draft, writing – review and editing. **Christine Hatté:** conceptualization, data curation, formal analysis, methodology, supervision, validation, writing – original draft, writing – review and editing. **Lauric Cécillon:** data curation, methodology. **Yannick Colin:** data curation, methodology. **Pierre Barré:** supervision, validation. **Claire Chenu:** supervision, validation. **Sophie Cornu:** methodology, supervision, validation, visualization, writing – original draft, writing – review and editing.

## Conflicts of Interest

The authors declare no conflicts of interest.

## Supporting information


**Appendix S1:** gcb70482‐sup‐0001‐AppendixS1.docx.

## Data Availability

The data that support the findings of this study are openly available in Zenodo at https://doi.org/10.5281/zenodo.16980274. This work is based on two global radiocarbon datasets from the International Soil Radiocarbon Database (ISRaD: https://doi.org/10.5281/zenodo.2613911) and the Laboratory of Climate and Environmental Sciences (LSCE: https://doi.org/10.14768/d9390f19‐228a‐443c‐98c3‐c2e58495d750).
