# Supplementary material for: Soil Carbon Dynamics Reshaped by Ancient Carbon Quantification
Source: Glob Chang Biol. 2025 Sep 2;31(9):e70482. doi: 10.1111/gcb.70482 (PMC12405676; doi:10.1111/gcb.70482)
Supplement: Supplementary file 1 — Appendix S1: gcb70482‐sup‐0001‐AppendixS1.docx. [file GCB-31-e70482-s001.docx]

**Supplementary Materials**

| **parent material**  **(literature)** | **Parent material**  **class (this paper)** | **profiles number** | **layers number** |
| --- | --- | --- | --- |
| loess | loess | 5 | 34 |
| loess to sedimentary rocks (carbonate) | NA | 2 | 12 |
| loess to sedimentary rocks (detrital) | NA | 1 | 5 |
| loess to volcanic rocks | NA | 14 | 78 |
| metamorphic rocks | metamorphic rocks | 12 | 54 |
| plutonic rocks | plutonic rocks | 15 | 94 |
| plutonic to metamorphic rocks | NA | 12 | 50 |
| plutonic, volcanic, metamorphic rocks | NA | 2 | 8 |
| sedimentary deposits (detrital) | sedimentary deposits | 14 | 139 |
| sedimentary deposits (lacustrine) | sedimentary deposits | 6 | 28 |
| sedimentary deposits (marine) | sedimentary deposits | 1 | 4 |
| sedimentary rocks (NA) | sedimentary rocks | 19 | 87 |
| sedimentary rocks (detrital to carbonate) | sedimentary rocks | 4 | 56 |
| sedimentary rocks (detrital) | sedimentary rocks | 27 | 200 |
| surficial deposits (glacial) | surficial deposits | 16 | 86 |
| surficial deposits (riverine to glacial) | surficial deposits | 2 | 10 |
| surficial deposits (riverine) | surficial deposits | 28 | 139 |
| surficial deposits (riverine, glacial, loess) | surficial deposits | 1 | 4 |
| volcanic rocks | volcanic rocks | 20 | 93 |

Table 1S: Parent material types used in this study derived from literature. All the parent material described as a mix are not considered (NA).

| **WRB**  **classification** | **profiles number** | **layers number** | **aOC ratio** | **Age_SOC_**  **(year)** | **Age_SOC<60 kyr_**  **(year)** |
| --- | --- | --- | --- | --- | --- |
| **Topsoils** |  |  |  |  |  |
| Albeluvisols | 4 | 11 | 0.16 ± 0.17 | 240 ± 390 | 80 ± 260 |
| Alisols/Acrisols | 30 | 58 | 0.11 ± 0.09 | 220 ± 510 | 7 ± 40 |
| Andosols | 13 | 25 | 0.13 ± 0.06 | 370 ± 530 | 80 ± 260 |
| Arenosols | 3 | 12 | 0.14 ± 0.05 | 0 ± 0 | 0 ± 0 |
| Calcisols | 1 | 4 | 0.09 ± 0.09 | 180 ± 240 | 0 ± 0 |
| Cambisols | 25 | 63 | 0.08 ± 0.09 | 310 ± 640 | 30 ± 150 |
| Cambisols/Regosols | 1 | 3 | 0.08 ± 0.03 | 540 ± 560 | 50 ± 80 |
| Chernozems | 11 | 40 | 0.06 ± 0.05 | 870 ± 970 | 530 ± 780 |
| Chernozems/Kastanozems/Phaeozems | 2 | 7 | 0.08 ± 0.04 | 250 ± 270 | 10 ± 30 |
| Cryosols | 7 | 14 | 0.21 ± 0.21 | 1240 ± 2060 | 240 ± 480 |
| Ferralsols | 39 | 128 | 0.10 ± 0.07 | 170 ± 440 | 30 ± 290 |
| Fluvisols | 1 | 3 | 0.05 ± 0.04 | 0 ± 0 | 0 ± 0 |
| Gleysols | 3 | 19 | 0.09 ± 0.08 | 80 ± 300 | 0 ± 0 |
| Histosols | 2 | 8 | 0.37 ± 0.23 | 2620 ± 2700 | 0 ± 0 |
| Kastanozems | 10 | 24 | 0.07 ± 0.04 | 600 ± 950 | 80 ± 190 |
| Luvisols | 23 | 94 | 0.11 ± 0.09 | 460 ± 1240 | 210 ± 1130 |
| Nitisols | 2 | 6 | 0.08± 0.02 | 240 ± 300 | 2 ± 5 |
| Phaeozems | 5 | 16 | 0.15 ± 0.06 | 310 ± 730 | 30 ± 120 |
| Planosols | 1 | 1 | 0.20 ± NA | 1810 ± NA | 40 ± NA |
| Plinthosols | 1 | 2 | 0.13 ± 0.08 | 330 ± 470 | 0 ± 0 |
| Podzols | 21 | 69 | 0.08 ± 0.08 | 360 ± 550 | 140 ± 390 |
| Regosols | 4 | 10 | 0.11 ± 0.08 | 210 ± 220 | 0 ± 0 |
| Solonetz | 1 | 3 | 0.03 ± 0.01 | 0 ± 0 | 0 ± 0 |
| Stagnosols | 2 | 7 | 0.10 ± 0.06 | 470 ± 620 | 100 ± 190 |
| Umbrisols | 6 | 13 | 0.09 ± 0.04 | 480 ± 490 | 150 ± 350 |
| Vertisols | 21 | 56 | 0.12 ± 0.09 | 410 ± 560 | 140 ± 390 |
| **Subsoils** |  |  |  |  |  |
| Albeluvisols | 4 | 9 | 0.45 ± 0.19 | 7150 ± 8110 | 3410 ± 4930 |
| Alisol/Acrisols | 30 | 76 | 0.26 ± 0.16 | 1630 ± 1870 | 100 ± 310 |
| Andosols | 13 | 33 | 0.34 ± 0.21 | 3170 ± 4230 | 670 ± 1930 |
| Arenosols | 3 | 12 | 0.21 ± 0.05 | 580 ± 550 | 0 ± 0 |
| Calcisols | 1 | 2 | 0.44 ± 0.11 | 1490 ± 30 | 0 ± 0 |
| Cambisols | 26 | 53 | 0.25 ± 0.19 | 1670 ± 1490 | 140 ± 330 |
| Cambisols/Regosols | 1 | 4 | 0.34 ± 0.26 | 1830 ± 520 | 10 ± 20 |
| Chernozems | 12 | 47 | 0.28 ± 0.19 | 2570 ± 2220 | 560 ± 840 |
| Chernozems/Kastanozems/Phaeozems | 2 | 5 | 0.18 ± 0.06 | 930 ± 130 | 0 ± 0 |
| Cryosols | 7 | 17 | 0.51 ± 0.29 | 6710 ± 5180 | 490 ± 890 |
| Ferralsols | 35 | 105 | 0.27 ± 0.15 | 1690 ± 1670 | 300 ± 910 |
| Fluvisols | 1 | 3 | 0.23 ± 0.03 | 1660 ± 1010 | 90 ± 150 |
| Gleysols | 3 | 29 | 0.21 ± 0.12 | 800 ± 2080 | 190 ± 630 |
| Histosols | 2 | 7 | 0.66 ± 0.33 | 12210 ± 9970 | 1110 ± 1400 |
| Kastanozems | 12 | 43 | 0.28 ± 0.15 | 2880 ± 2080 | 470 ± 870 |
| Luvisols | 23 | 157 | 0.30 ± 0.18 | 2480 ± 2870 | 530 ± 2010 |
| Nitisols | 2 | 11 | 0.18 ± 0.06 | 1290 ± 1430 | 330 ± 520 |
| Phaeozems | 7 | 19 | 0.28 ± 0.15 | 2380 ± 2740 | 430 ± 750 |
| Planosols | 3 | 8 | 0.40 ± 0.17 | 3640 ± 2050 | 220 ± 640 |
| Plinthosols | 1 | 1 | 0.61 ± NA | 6430 ± NA | 0 ± NA |
| Podzols | 20 | 41 | 0.20 ± 0.17 | 1140 ± 1170 | 120 ± 280 |
| Regosols | 4 | 7 | 0.17 ± 0.07 | 740 ± 400 | 20 ± 50 |
| Solonetz | 1 | 5 | 0.14 ± 0.07 | 430 ± 730 | 180 ± 400 |
| Stagnosols | 2 | 5 | 0.29 ± 0.20 | 2490 ± 1390 | 320 ± 450 |
| Umbrisols | 6 | 26 | 0.19 ± 0.09 | 2080 ± 720 | 550 ± 740 |
| Vertisols | 25 | 110 | 0.23 ± 0.16 | 1910 ± 1090 | 400 ± 790 |
| **Deepsoils** |  |  |  |  |  |
| Alisols/Acrisols | 3 | 5 | 0.47 ± 0.07 | 2600 ± 1490 | 0 ± 0 |
| Andosols | 1 | 1 | 0.61 ± NA | 2410 ± NA | 0 ± NA |
| Arenosols | 2 | 2 | 0.36 ± 0.06 | 2270 ± 580 | 0 ± 0 |
| Calcisols | 1 | 1 | 0.22 ± NA | 2000 ± NA | 10 ± NA |
| Cambisols | 8 | 10 | 0.59 ± 0.22 | 3530 ± 1820 | 20 ± 80 |
| Chernozems | 11 | 20 | 0.59 ± 0.27 | 7890 ± 2820 | 1220 ± 1910 |
| Cryosols | 1 | 1 | 0.62 ± NA | 8270 ± NA | 590 ± NA |
| Ferralsols | 30 | 51 | 0.50 ± 0.22 | 4640 ± 2960 | 330 ± 1070 |
| Fluvisols | 1 | 1 | 0.54 ± NA | 2820 ± NA | 0 ± NA |
| Gleysols | 1 | 1 | 0.37 ± NA | 0 ± NA | 0 ± NA |
| Kastanozems | 8 | 25 | 0.65 ± 0.24 | 6980 ± 2960 | 300 ± 510 |
| Luvisols | 13 | 67 | 0.52 ± 0.20 | 4700 ± 2590 | 830 ± 2550 |
| Nitisols | 2 | 6 | 0.30 ± 0.07 | 2430 ± 2070 | 770 ± 1190 |
| Phaeozems | 3 | 12 | 0.51 ± 0.18 | 5370 ± 2250 | 1090 ± 1220 |
| Planosols | 3 | 3 | 0.57 ± 0.20 | 8650 ± 1050 | 1990 ± 2030 |
| Plinthosols | 1 | 1 | 0.83 ± NA | 10 760 ± NA | 0 ± NA |
| Podzols | 1 | 7 | 0.21 ± 0.16 | 2480 ± 1740 | 800 ± 770 |
| Stagnosols | 1 | 2 | 0.75 ± 0.14 | 4730 ± 810 | 0 ± 0 |
| Umbrisols | 5 | 17 | 0.54 ± 0.23 | 5330 ± 2390 | 370 ± 860 |
| Vertisols | 20 | 111 | 0.44 ± 0.20 | 5200 ± 2820 | 890 ± 2010 |

Table 2S: AgeS_OC_ and corrected age (Age_SOC<60 kyr_) at different depths for the whole studied soil groups from the WRB classification. Number of profiles and analyzed layers are given as their respective aOC ratio.

| **parent material** | **profile**  **number** | **layer number** | **aOC.ratio** | **ageSOC** | **ageSOC_>60 ky_** |
| --- | --- | --- | --- | --- | --- |
| **topsoil (0-30 cm)** |  |  |  |  |  |
| loess | 4 | 12 | 0.05 ± 0.03 | 1 450 ± 990 | 430 ± 510 |
| metamorphic rocks | 12 | 21 | 0.10 ± 0.06 | 470 ± 620 | 100 ± 280 |
| plutonic rocks | 15 | 40 | 0.08 ± 0.06 | 220 ± 320 | 35 ± 140 |
| sedimentary deposits | 21 | 64 | 0.12 ± 0.11 | 190 ± 390 | 55 ± 210 |
| sedimentary rocks | 50 | 118 | 0.13 ± 0.11 | 410 ± 980 | 55 ± 250 |
| surficial deposits | 46 | 117 | 0.11 ± 0.11 | 320 ± 550 | 45 ± 240 |
| volcanic rocks | 20 | 44 | 0.10 ± 0.09 | 320 ± 700 | 30 ± 130 |
| **subsoil (30-100 cm)** |  |  |  |  |  |
| loess | 5 | 16 | 0.22 ± 0.19 | 3 350 ± 1 390 | 870 ± 930 |
| metamorphic rocks | 12 | 32 | 0.24 ± 0.22 | 2 410 ± 4 140 | 540 ± 1 880 |
| plutonic rocks | 15 | 44 | 0.23 ± 0.17 | 1 330 ± 1 480 | 110 ± 310 |
| sedimentary deposits | 20 | 64 | 0.26 ± 0.16 | 2 180 ± 3 710 | 750 ± 2 160 |
| sedimentary rocks | 50 | 157 | 0.28 ± 0.16 | 2 460 ± 3 180 | 310 ± 640 |
| surficial deposits | 47 | 104 | 0.28 ± 0.18 | 2 200 ± 2 410 | 300 ± 1 010 |
| volcanic rocks | 20 | 49 | 0.28 ± 0.19 | 2 070 ± 2 810 | 230 ± 700 |
| **deepsoil (>100 cm)** |  |  |  |  |  |
| loess | 4 | 6 | 0.35 ± 0.27 | 6 210 ± 2 670 | 1 670 ± 2 260 |
| metamorphic rocks | 1 | 1 | 0.61 ± NA | 2 410 ± NA | 0 ± NA |
| plutonic rocks | 4 | 10 | 0.37 ± 0.16 | 3 050 ± 2 180 | 480 ± 960 |
| sedimentary deposits | 7 | 43 | 0.40 ± 0.17 | 4 900 ± 3 010 | 1 270 ± 2 620 |
| sedimentary rocks | 19 | 68 | 0.54 ± 0.25 | 4 880 ± 2 440 | 260 ± 590 |
| surficial deposits | 13 | 18 | 0.40 ± 0.22 | 5 730 ± 3 660 | 1 860 ± 2 530 |

Table 3S: AgeS_OC_ and corrected age (Age_SOC<60 kyr_) at different depths for the selected parent materials. Number of profiles and analyzed layers are given as their respective aOC ratio.

| **WRB soils groups** | **profils number** | **layers number** | **surface**  **(Mkm^2^)** |
| --- | --- | --- | --- |
| albeluvisol | 4 | 11 | 3.20 |
| **Alisol/Acrisols** | **30** | **58** | **1.00** |
| **Andosols** | **13** | **25** | **1.10** |
| Arenosols | 3 | 12 | 9.03 |
| Calcisols | 1 | 4 | 10.00 |
| **Cambisols** | **25** | **63** | **10.00** |
| Cambisols/Regosols | 1 | 3 | n.c. |
| **Chernozems** | **11** | **40** | **2.30** |
| Chernozems/Kastanozems/Phaeozems | 2 | 7 | n.c |
| **Cryosols** | **7** | **14** | **18.00** |
| **Ferralsols** | **39** | **128** | **7.50** |
| Fluvisols | 1 | 3 | 3.50 |
| Gleysols | 3 | 19 | 7.20 |
| Histosols | 2 | 8 | 3.50 |
| **Kastanozems** | **10** | **24** | **4.65** |
| **Luvisols** | **23** | **94** | **6.00** |
| Nitisols | 2 | 6 | 2.00 |
| **Phaeozems** | **5** | **16** | **1.90** |
| Planosols | 1 | 1 | 1.30 |
| Plinthosols | 1 | 2 | 0.60 |
| **Podzols** | **21** | **69** | **4.85** |
| Regosols | 4 | 10 | 2.60 |
| Solonetz | 1 | 3 | 1.35 |
| Stagnosols | 2 | 7 | 1.75 |
| **Umbrisols** | **6** | **13** | **1.00** |
| **Vertisols** | **21** | **56** | **3.35** |
| Total soils surface (26 soils groups) |  |  | 107.68 |
| Total soils surface (12 soils groups) |  |  | 66.65 |

Table 4S: studied soils groups from the two databases with their respective total land surface they cover. in bold: soils groups where the number of profiles over than 4 and meeting our conditions for our analyses (parent materials). Without permanent snow and ice, the total land surface is set at 129 Mkm^2^ (www.isqper-is.eu, www.isric.org).

Figure 1S: linear modelling (F^14^C.SOC=f(SOC)) with coefficient correlations of the WRB soil groups with two examples (when it is possible): one with high R^2^ and another one with low R^2^. Most of the studied soil profiles, (i.e. 87%) exhibit a coefficient correlation over than 0.90 and was selected for the calculation of aOC mass concentrations in top, sub and deepsoils.
